# Organ Donation After Euthanasia in Patients Suffering From Psychiatric Disorders: 10-Years of Preliminary Experiences in the Netherlands

**DOI:** 10.3389/ti.2023.10934

**Published:** 2023-02-09

**Authors:** Nathalie van Dijk, Paulan Stärcke, Wim de Jongh, Nichon Jansen, David Shaw, Jan Bollen, Walther van Mook

**Affiliations:** ^1^ Department of Intensive Care Medicine, Maastricht University Medical Center+, Maastricht, Netherlands; ^2^ Euthanasia Expertise Center, The Hague, Netherlands; ^3^ Department of Surgery, Transplantation Coordination Services, Maastricht University Medical Center+, Maastricht, Netherlands; ^4^ Dutch Transplant Foundation, Leiden, Netherlands; ^5^ Care and Public Health Research Institute, Maastricht University, Maastricht, Netherlands; ^6^ Institute for Biomedical Ethics, University of Basel, Basel, Switzerland; ^7^ Department of Anesthesiology, Pain and Palliative Medicine, Radboud University Medical Center, Nijmegen, Netherlands; ^8^ Academy for Postgraduate Training, Maastricht University Medical Center+, Maastricht, Netherlands; ^9^ School of Health Professions Education, Maastricht University, Maastricht, Netherlands

**Keywords:** organ donation, euthanasia, psychiatric illness, disorder, suffering

## Abstract

Euthanasia based on psychiatric suffering, followed by subsequent organ donation, is considered medically and legally permissible in the Netherlands. Although organ donation after euthanasia (ODE) in patients suffering from unbearable psychiatric illness is performed, it is not specifically addressed in the Dutch guideline on organ donation after euthanasia, and national data on ODE in psychiatric patients have not yet been published. In this article, the preliminary results of the 10-year Dutch case series of psychiatric patients who choose ODE are presented and potential factors influencing opportunities for donation in this population are discussed. We conclude that further future in-depth qualitative exploration of ODE in patients suffering from psychiatric illness and its associated ethical and practical dilemmas, including the consequences for the patient and their family and healthcare professionals, will be important to help make sense of potential barriers to donation for people undergoing euthanasia as a result of psychiatric suffering.

## Graphical Abstract

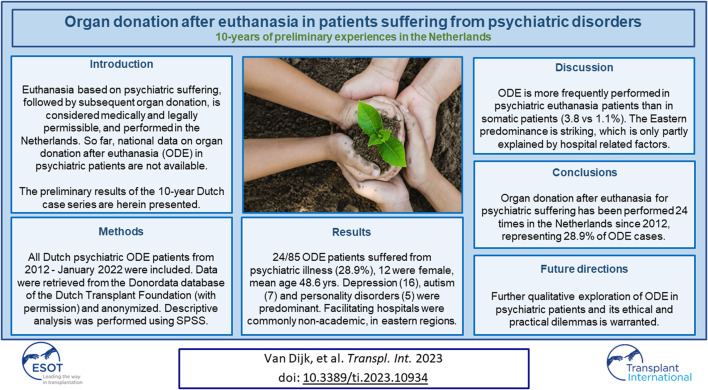

## Introduction

### Euthanasia

Physician assisted dying makes it possible for a patient to die a self-chosen, peaceful death. Euthanasia, where a physician administers a lethal drug intravenously to a patient, is currently legally permitted in Belgium, the Netherlands, Luxemburg, Colombia, Canada and parts of Australia (1, 2). Recently, euthanasia was allowed in Spain since June 2021 and in New Zealand since November 2021. In the Netherlands, the societal, judicial, ethical and medical debate resulted in the Termination of Life on Request and Assisted Suicide Act in 2002. Since its legalization, euthanasia has been allowed following a voluntary request if the mentally competent patient is suffering hopelessly, irreversibly and unbearably, while no other reasonable options are available, and only after a second, independent physician is consulted. 82,963 patients underwent euthanasia between 2002 and 2021 in the Netherlands, most commonly because of end-stage cancer (3). In general, the patient requests euthanasia *via* his general practitioner.

In 1994, a Dutch psychiatrist facilitated physician assisted suicide in a patient with underlying psychiatric suffering for the first time, in the so-called Chabot case. The Dutch supreme court reasoned that unbearable and irreversible psychiatric suffering could justify physician assisted death, but mandated consultation of a second independent psychiatrist in future cases (4). Although extra due diligence criteria for patients with a psychiatric cause of suffering are not included in the 2002 Termination of Life on Request and Assisted Suicide Act, they are formulated in the specific guideline by the Dutch Society of Psychiatrists, and currently applied in practice: if the cause of suffering is psychiatric in nature, either the treating physician or the second independent physician needs to be a psychiatrist, and a second opinion by a psychiatrist competent to assess the patient’s specific pathology is mandatory (5).

It should be underscored that euthanasia in psychiatric suffering is only allowed when the patient has maximally pursued all reasonably possible treatments. Consequently, it takes several years before a euthanasia request is granted. This time period is essential to assess whether previously attempted treatments were (in)efficacious, to explore potential new medical treatment strategies, and to evaluate the patient’s perseverance. Ultimately, an estimated 10% of euthanasia requests in psychiatric patients is granted (6).

Every euthanasia case is reviewed by a Regional Euthanasia Review Committee consisting of a lawyer, an ethicist and a doctor 6 weeks after the procedure, supported and advised by a secretary (also a lawyer). While a psychiatrist is required at the earlier stage of assessment for euthanasia, this is not the case in the *post hoc* review of the eventual procedure. If the review committee determines that the due diligence requirements were not fulfilled, the performing physician can be prosecuted. The additional requirements for patients suffering from a psychiatric illness formulated in the Dutch Society of Psychiatrists guideline are also used in the *post hoc* analysis (see below).

In 2021, 7666 patients underwent euthanasia in the Netherlands, of whom 115 (1.5%) suffered primarily from a psychiatric disease. Since 2002, *post hoc* analysis confirmed procedural correctness in all cases, and prosecution in cases in which psychiatric suffering was the basis for the euthanasia request, has so far not occurred.

### Organ Donation After Euthanasia (ODE)

 Organ Donation after Euthanasia is currently being performed within legal boundaries in Belgium, the Netherlands, Canada and Spain. Neither the Dutch law on organ donation nor Dutch Termination of Life on Request and Assisted Suicide Act preclude organ donation after euthanasia, although these laws were drafted independently. The subject of organ donation can only be raised by the patient after the euthanasia request is approved. This sequential order, carefully laid down in the National Dutch Organ Donation after Euthanasia guideline (7) aims to ensure the separation of the euthanasia assessment procedure and subsequent organ donation request, and to prevent any influence that the euthanasia request could have on the organ donation request, and *vice versa*. In case a patient raises the issue of organ donation prematurely, the treating physician should postpone discussing the topic until after the euthanasia assessment procedure has been correctly completed, and the euthanasia requests has been granted. If the patient however does not raise the issue of organ donation, the physician is legally obligated to check the patient’s registration in the Dutch donor registry, in which patient preferences regarding donation are newly documented since the introduction of the national Dutch op-out system in July 2020 (8). In the absence of a patient’s registered active refusal to donate, the patient’s presumed consent may scaffold further dialogue facilitating shared decision making and respecting the patient’s autonomy regarding organ donation after euthanasia.

Organ donation after euthanasia is a donation after circulatory death (DCD) procedure (9). DCD is possible in the absence of medical contraindications, and after fulfilling all criteria in the Dutch Organ Donation Act, including the guideline regarding death determination by the Dutch Health Council (10). The most common general contraindication for organ donation is malignancy. Of all patients who underwent euthanasia an estimated 10% are potentially medically eligible to donate their organs (11). Most commonly these patients suffer from underlying neurodegenerative diseases such as amyotrophic lateral sclerosis (ALS) and Huntington’s disease (11). From 2012, when the first ODE was performed, to January 2022, 85 patients donated their organs following euthanasia in the Netherlands. Following DCD donation, it is possible to donate lungs, kidneys, pancreas and liver (12). In March 2021, DCD heart donation has been introduced in the Netherlands (13), which recently resulted in the first heart donated by a patient suffering from an unbearable psychiatric disease who requested for euthanasia, which was subsequently successfully transplanted. The euthanasia procedure is most commonly performed in an intensive care, medium care or post anesthesia care unit by the treating physician (14). This environment can facilitate distantly monitoring the patient using an arterial line, and these units are often located a short distance from the operation theatre, which helps limiting ischemic damage to the procured organs. Comparable to the strict separation of the euthanasia and organ donation requests procedures, the transplantation procedure is strictly separated from the organ donation procedure, to avoid any conflict of interest. More detailed information about the practical aspects and governance of ODE has been published previously (7).

Currently, organ donation after euthanasia in patients suffering from unbearable psychiatric illness is mentioned, yet not addressed in detail, in the Dutch guideline on organ donation, presumably because of the perceived limited occurrence and unfamiliarity with ODE requests in this category of patients.

So far, experiences with organ donation after euthanasia in patients suffering from a psychiatric disease are limited (14) and not previously systematically explored in the literature. In this article, in order to identify temporal trends in organ donation after euthanasia, an overview of the preliminary data of *all* cases of organ donation after euthanasia due to psychiatric suffering in the Netherlands is presented, and compared to data on euthanasia and ODE in somatic patients, followed by a review of the practical hurdles patients and healthcare professionals may encounter when they encounter a request for this specific combined procedure.

The aim of this study is to increase the understanding of both euthanasia in patients with psychiatric suffering and its combination with organ donation.

## Patients and Methods

All patients who underwent organ donation after euthanasia due to a psychiatric illness from 2012 until January 2022 in the Netherlands were included in the analysis. Data on gender, age, and underlying psychiatric disorder, as well as the facilitating hospitals were retrieved from the Donordata database of the Dutch Transplant Foundation database (with permission) and anonymized. Retrospective analysis of prospectively collected data were performed with descriptive analysis using SPSS version 26.0 (15).

## Results

This section will subsequently discuss the eligibility for euthanasia and organ donation, the background of the euthanizing physicians, the patients’ underlying psychiatric conditions, the types of facilitating hospitals, the geographical distribution of the ODE procedures, and the nature and number of organs transplanted resulting from ODE procedures in patients with underlying psychiatric disorders in the Netherlands.

### Eligibility for Euthanasia and Organ Donation

Over the ten-year study period 2012–2021 59,546 patients underwent euthanasia of whom 58,912 suffered from a somatic disorder. The number of patients that underwent euthanasia for an underlying psychiatric disorder was 634 (1.1%). An estimated 10% (5955) of patients who undergo euthanasia in general are medically eligible to donate one or more organs (11).

An estimated 5321 (5955–634) somatic patients would theoretically be eligible for organ donation, and 61 actually donated after euthanasia (61/5321; 1.1%). Assuming contraindications such as malignancy are absent, theoretically, all patients who undergo euthanasia for psychiatric reasons are medically eligible to donate their organs. The percentage of patients with an underlying psychiatric disorder that underwent organ donation after euthanasia (24/634; 3.8%) in perspective of the percentage of patients who donated after euthanasia based on an underlying somatic disorder, is thus high.

Since 2012, 85 patients underwent organ donation after euthanasia, of which 24 (28.9%) because of psychiatric suffering and 61 because of somatic suffering (see Table 1).

**TABLE 1:** Evolution of ODE in patients suffering from somatic respectively psychiatric disorders over time.

Year	2012	2013	2014	2015	2016	2017	2018	2019	2020	2021	Total
Somatic disorders	1	2	2	9	7	11	9	8	5	7	61
Psychiatric disorders		1			1	2	3	5	7	5	24

In the 24 cases, there was an equal division in gender, with an average age of 48.6 (range 21–72) years.

### Euthanising Physicians

The physicians performing euthanasia in the procedures related to psychiatric suffering were physicians from the Dutch Euthanasia Expertise Centre in half the cases (13), followed by psychiatrists (6), general practitioners (4) and specialist in elderly care (specialist in geriatric care e.g. geriatric patients in nursing homes) (1).

### Underlying Psychiatric Conditions

The underlying psychiatric conditions, as documented in, and retrieved from the database, were personality disorders, depression, autism, posttraumatic stress disorders (PTSS), and Attention Deficit Hyperactivity Disorder (ADHD). Depression (16 pts), autism (7 pts) and personality disorders (5 pts) were predominant. In 2 cases a combination of a somatic disorder with a psychiatric disorder was reported. In 11 cases a combination of psychiatric disorders was documented.

### Types of Facilitating Hospitals

The procedures in patients with underlying psychiatric disorders was performed in 4 out of the total of seven university medical centers in 9 out of 24 cases, and affiliated hospitals in the others. The four university medical centers facilitated the procedures 4, 3, 1 and 1 times, respectively. One affiliated hospital performed the procedure four times, another affiliated hospital three times, and two hospitals two times. Four affiliated hospitals performed the procedure once.

### Geographical Distribution

Geographically, the facilitating hospitals were predominantly located in the Eastern part of the Netherlands. Nearly all euthanasia cases were performed in a medium or intensive care setting. The remaining 2 cases in an outpatient treatment (day care) setting, respectively post anesthesia care setting. All cases were facilitated by intensivists.

### Organs Transplanted

The 24 cases lead to 107 (mean 4.5, range 1–7) organ transplantations. The transplanted organs were kidneys (46), lungs (35), liver (16), pancreas (5) and heart (2).

## Discussion

Until January 1st 2022, 24 patients suffering from an underlying psychiatric disorder have chosen to donate their organs following euthanasia (from a total of 85, 27%). The primary goal of organ donation after euthanasia is to facilitate the patient’s last wish. The positive consequences for the transplant waiting lists are subordinate. Nevertheless, the preliminary results of this unique study do demonstrate that patients with psychiatric suffering who underwent ODE have improved and/or extended the lives of dozens of patients who were on the waiting list for an organ transplantation.

Since 2012 the incidence of ODE due to somatic disorders peaked in 2017 (in which 11 cases were documented), where after it more or less remained stable (see Table 1). The first case of ODE in a patient suffering from a psychiatric disorder was reported in 2013, and the number of cases annually seems to increase since 2016. In 2020 the number of patients that underwent ODE for psychiatric disease was higher than that for somatic disorders. Over recent years there has been increasing interest in patients’ journeys as illustrated by publications in the non-scientific, popular media. It is, however, difficult to predict whether the number of requests for ODE will consequently potentially increase (17).

A remarkably higher proportion of patients with psychiatric conditions donated their organs than among patients who sought euthanasia for other reasons. Over the 10 years study period 2012–2021 59,546 patients underwent euthanasia of which 58,912 suffered from a somatic disorder, the number of patients that underwent euthanasia for an underlying psychiatric disorder was 634 (1.1%). An estimated 10% (5955) of patients who undergo euthanasia in general are medically eligible to donate one or more organs (11). We assume that all patients who undergo euthanasia for psychiatric reasons are medically eligible to donate their organs. An estimated 5321 (5955-634) somatic patients would theoretically be eligible for organ donation, and 61 actually donated after euthanasia (61/5321; 1.1%). The percentage of patients with an underlying psychiatric disorder that underwent organ donation after euthanasia (24/634; 3.8%) in perspective of the percentage of patients who donated after euthanasia based on an underlying somatic disorder is thus remarkably high.

In addition, patients with ODE based on an underlying psychiatric disease were 5 years younger than the average population of patients who underwent ODE (48.8 years versus 53.8 years (latter data are not shown). Patients who undergo organ donation after euthanasia appear to be younger than the general euthanasia population, although an average age for the latter group is not reported (18). However, the vast majority of patients in 2020 and 2021 (87.6% resp. 89%) was reportedly aged over 60 (17, 18).

*How can these results be interpreted and what do they mean for the practice of organ donation after euthanasia?*

It should be acknowledged that the analysis herein presented represents the willingness to donate of a small subgroup of psychiatric patients whose euthanasia and donation requests were both granted. A recent 2019 study of a stratified sample of 5361 non-sudden deaths from the central Dutch Registry of Statistics revealed that 3.4% had a psychiatric disorder (16). The frequency of euthanasia and assisted suicide (EAS) requests was 11.4%, compared to 11.2% in the whole population, and 8% among people with an accumulation of health problems (16).

Six percent of all deceased patients actually received EAS. This percentage was lower among psychiatric patients (4.8%). In case of a psychiatric disorder, the presence of (severe) symptoms other than pain (75.4%) and expected suffering (53.5%) were important reasons to grant EAS. Across the full sample, the two most important reasons to grant the request were the lack of prospect of improvement (81.9%–94.6%) and the autonomy of the patient (72.4%–85.8%) (16).

The main reason for refusal of EAS among *all* deceased patients, was death of the patients before the request was granted. The most important reason to refuse the request in psychiatric patients was that the due diligence criteria were not met, particularly regarding the well-considered nature of the request (16).

Comparison of donation rates in the general group of patients who underwent euthanasia with patients who underwent euthanasia due to psychiatric suffering is interesting. It can potentially answer the question regarding whether consent for donation and actual donation rates were higher in the euthanasia due to psychiatric suffering group, and thus whether rates to withdraw consent for either the euthanasia or organ donation procedure. However, data on donation consent and subsequent withdrawal rates in psychiatric versus non-psychiatric patients in a larger population than herein presented are unavailable, to the best of our knowledge.

One can thus only speculate on the reasons contributory to the herein observed differences in donation after euthanasia in somatic versus psychiatric patients. Possible explanations may be that psychiatric patients, for example compared to patients suffering from ALS, are physically more able to gather information, e.g. on the internet, on the possibilities of organ donation after euthanasia, or that they are more physically able to undergo the preparatory examinations necessary for donation. Another possibility is that patients suffering from psychiatric illness are more altruistic, or have become more altruistic, due to reflections about their own lives, or experiences with organ donation in their surroundings, and consequently want to finalize their lives with an altruistic gift to, for them unknown others (17). Theoretically, the physicians performing the euthanasia could also more frequently raise the issue of organ donation after euthanasia in psychiatric patients than somatic patients. However, the Dutch ODE guideline advises against proactively raising the option of donation after euthanasia in general (14). In addition, greater caution regarding competency is warranted and attributed in patients with mental conditions.

Furthermore, it is possible that psychiatric patients assume, more frequently than somatic patients, that their organs will be medically suitable to donate organs (see below). Based on their relatively young age and underlying psychiatric disorders (in comparison with patients with underlying somatic disorders), it indeed might be assumed that these patients have healthy organs that are more likely to be suitable for transplantation. Although 107 organs were successfully transplanted from these 24 donors, it is however premature to draw firm conclusions whether the organs of patients with underlying psychiatric disorders are indeed in better condition. In contrast to the general population, people with mental disorders for example have high rates of adverse health behaviors, including tobacco smoking, substance use, physical inactivity, and poor diet, e.g. resulting in cardiovascular disorders and diabetes (20). Although the preliminary results of transplantation following organ donation after euthanasia *in general* demonstrate good functional results (21,22,23), further research on the transplantation results following organ donation after euthanasia in psychiatric suffering still has to be performed.

Despite occurring relatively frequently, organ donation after euthanasia in psychiatric suffering is nevertheless surrounded by several mainly ethical challenges, related primarily to the euthanasia, such as the important issue of competency relating to both the euthanasia and organ donation requests, but also to the subsequent organ donation procedure in these patients, such as the impact on the health professionals involved in the procedures. Discussing these challenges is beyond the scope of this article.

In summary, patients can suffer unbearably and hopelessly from a psychiatric disease, comparable to patients with physical underlying disorders. Any request for euthanasia should be carefully considered, with particular attention paid to the patient’s decision-making capacity, given the context of psychiatric issues. Any subsequent wish to donate organs is an extremely altruistic act, should likewise be subject of careful and deliberate consideration, critically considering any conflicts of interest. The strict separation between the euthanasia and organ donation requests on the one hand, and the organ donation and transplantation stages, with different physicians involved in the different stages on the other, aims to prevent such conflicts of interest. The effects of the requested organ donation on the transplantation waiting lists are principally irrelevant in this regard.

This study has strengths but also several limitations. The study is the first to provide a unique, preliminary insight into ODE in patients with psychiatric illness. The data reported here are however limited to the data formally accessible for the research group. The granularity of the data is thus limited. The information as retrieved from the database, e.g. the categorization of psychiatric illness in several cases, was not debated by the researchers. Somatoform complaints not primarily documented as a psychiatric disease in the database are thus not represented in this current case series. More detailed information on the psychiatric illness, treatments attempted and the duration of both illness and treatment was not available in the database.

Although the Eastern geographical predominance of ODE in psychiatric illness is striking, data which may provide insights into the reasons for this observation are likewise not available. It is known that not all hospitals in the Netherlands are willing to honor a patient’s wish for organ donation after euthanasia. Such local and regional differences may perhaps at least partly explain some of the observed geographical differences. A recent cross sectional study on the crude rates of euthanasia (not followed by organ donation) indeed revealed considerable geographical variation across the Netherlands (24), however with a seemingly Western predominance. Associated factors herein were age, church attendance, political orientation, income, self-experienced health and availability of voluntary workers. After adjustment for these characteristics a considerable amount of geographical variation remained, which also calls for further exploration.

The preliminary results of this study nevertheless provide sufficient anchors for further qualitative exploratory interview studies among the different healthcare professionals involved and perhaps the patients’ relatives to elucidate the reasons underlying some of the findings in this study. Herein their experiences regarding the timing and initiation of the donation request by patients and/or physicians, the use and usefulness of potential familiarization and/or exploration meetings between the patient and the facilitating hospital’s staff, the option to start the ODE procedure from home (23–25), and the emotional burden for patients and their relatives to die in hospital(14), as well as the emotional burden of healthcare professionals facilitating the procedures can be explored more in depth. In addition, the transplantation results of the donated organs necessitate further short-, mid- and long-term follow up. Such follow up studies are currently performed.

In conclusion: euthanasia because of psychiatric suffering, followed by organ donation, is medically and legally permissible and performed in Dutch healthcare practice. The preliminary results of this 10-year Dutch case series contribute to increasing the understanding of both euthanasia in patients with psychiatric suffering and its combination with organ donation. Organ donation after euthanasia in psychiatric suffering has been performed 24 times in the Netherlands since 2012, representing 28.9% of cases. So far, this patient category is not specifically addressed in the national Dutch guideline on organ donation, since the ethical debate hereon is ongoing and data on ODE were so far completely lacking. This specific type of organ donation after euthanasia, and its associated ethical and practical dilemmas, including competency and consent issues, as well the consequences for the patient’s family and healthcare professionals, necessitate further careful qualitative exploration before this topic can potentially become part of a revised version of the existing national guideline on organ donation after euthanasia.

## Data Availability

The original contributions presented in the study are included in the article/supplementary material, further inquiries can be directed to the corresponding author.
